# Klebsiella pneumoniae Carbapenemase (KPC)-Producing K. pneumoniae at a Single Institution: Insights into Endemicity from Whole-Genome Sequencing

**DOI:** 10.1128/AAC.04292-14

**Published:** 2015-02-11

**Authors:** Amy J. Mathers, Nicole Stoesser, Anna E. Sheppard, Louise Pankhurst, Adam Giess, Anthony J. Yeh, Xavier Didelot, Stephen D. Turner, Robert Sebra, Andrew Kasarskis, Tim Peto, Derrick Crook, Costi D. Sifri

**Affiliations:** a Division of Infectious Diseases and International Health, Department of Medicine, University of Virginia Health System, Charlottesville, Virginia, USA; b Clinical Microbiology, Department of Pathology, University of Virginia Health System, Charlottesville, Virginia, USA; c Modernizing Medical Microbiology Consortium, Nuffield Department of Clinical Medicine, John Radcliffe Hospital, Oxford University, Oxford, United Kingdom; d Department of Infectious Disease Epidemiology, School of Public Health, Imperial College London, London, United Kingdom; e Department of Public Health Sciences, School of Medicine, University of Virginia, Charlottesville, Virginia, USA; f Icahn Institute and Department of Genetics and Genomic Sciences, Icahn School of Medicine, Mount Sinai, New York, New York, USA; g Public Health England, Microbiology Services, London, United Kingdom; h Office of Hospital Epidemiology, University of Virginia Health System, Charlottesville, Virginia, USA

## Abstract

The global emergence of Klebsiella pneumoniae carbapenemase-producing K. pneumoniae (KPC-*Kp*) multilocus sequence type ST258 is widely recognized. Less is known about the molecular and epidemiological details of non-ST258 K. pneumoniae in the setting of an outbreak mediated by an endemic plasmid. We describe the interplay of *bla*_KPC_ plasmids and K. pneumoniae strains and their relationship to the location of acquisition in a U.S. health care institution. Whole-genome sequencing (WGS) analysis was applied to KPC-*Kp* clinical isolates collected from a single institution over 5 years following the introduction of *bla*_KPC_ in August 2007, as well as two plasmid transformants. KPC-*Kp* from 37 patients yielded 16 distinct sequence types (STs). Two novel conjugative *bla*_KPC_ plasmids (pKPC_UVA01 and pKPC_UVA02), carried by the hospital index case, accounted for the presence of *bla*_KPC_ in 21/37 (57%) subsequent cases. Thirteen (35%) isolates represented an emergent lineage, ST941, which contained pKPC_UVA01 in 5/13 (38%) and pKPC_UVA02 in 6/13 (46%) cases. Seven (19%) isolates were the epidemic KPC-*Kp* strain, ST258, mostly imported from elsewhere and not carrying pKPC_UVA01 or pKPC_UVA02. Using WGS-based analysis of clinical isolates and plasmid transformants, we demonstrate the unexpected dispersal of *bla*_KPC_ to many non-ST258 lineages in a hospital through spread of at least two novel *bla*_KPC_ plasmids. In contrast, ST258 KPC-*Kp* was imported into the institution on numerous occasions, with other *bla*_KPC_ plasmid vectors and without sustained transmission. Instead, a newly recognized KPC-*Kp* strain, ST941, became associated with both novel *bla*_KPC_ plasmids and spread locally, making it a future candidate for clinical persistence and dissemination.

## INTRODUCTION

Due to the lack of available therapeutic options, the Centers for Disease Control and Prevention (CDC) listed carbapenem-resistant members of the family Enterobacteriaceae as an “immediate public health threat that requires urgent and aggressive action” (1). Dissemination of carbapenem-resistant Enterobacteriaceae in the United States has largely been due to a serine class A β-lactamase called Klebsiella pneumoniae carbapenemase (KPC) (2). Although the *bla*_KPC_ gene commonly resides in mobile plasmids that can move freely between genera of Enterobacteriaceae (3, 4), *bla*_KPC_ plasmids have become predominantly associated with KPC-producing K. pneumoniae (KPC-*Kp*), particularly multilocus sequence type ST258 (5–10) and other highly related strains in the clonal complex of ST258 (11, 12).

The *bla*_KPC_ gene is mostly described as being contained within Tn*4401*, a 10-kb Tn*3* family transposon capable of mobilization through transposition (13). This transposon has been found inserted in a variety of plasmids in ST258 KPC-*Kp* (6, 14–17). However, less is known about the diversity of *bla*_KPC_ plasmids participating in the wider dissemination of carbapenem resistance among other lineages of K. pneumoniae and among other species of Enterobacteriaceae. The few studies that have been undertaken are limited by the restricted extent of plasmid relationships and host bacterial phylogeny investigated. Consequently, the associations between host strains and/or between plasmids could not be fully elucidated (4, 18, 19).

Whole-genome sequencing (WGS) has been shown to resolve transmission networks of the nosocomial pathogens Clostridium difficile, K. pneumoniae, and Staphylococcus aureus (20–24). It has also been instrumental in disentangling the phylogeographic spread of key pathogens such as Mycobacterium tuberculosis, Escherichia coli, Salmonella enterica, Vibrio cholerae, methicillin-resistant S. aureus, and Streptococcus pneumoniae (23–29). The use of WGS for investigating the emergence and dissemination of resistance among a prospectively collected population of K. pneumoniae bacteria within a single institution and their plasmids has not yet been reported.

We used WGS to investigate the molecular epidemiology of KPC-*Kp* occurring in the setting of an outbreak mediated by an endemic plasmid in a single hospital ecosystem over a 5-year period, dating from introduction of both a KPC-*Kp* and a KPC-producing Klebsiella oxytoca (KPC-*Ko*) by the first observed case in 2007 (30, 31). This study provides new insights into the dynamic relationships between K. pneumoniae host strains and plasmids involved in the emergence and spread of plasmid-mediated antibiotic resistance.

(This work was originally presented in part in abstract format as a poster at the 24th European Congress of Clinical Microbiology and Infectious Diseases [ECCMID] in Barcelona, Spain, 10 to 13 May 2014.)

## MATERIALS AND METHODS

### Isolate collection, characterization, and selection for WGS.

Isolates were prospectively collected from August 2007 to September 2012 through the Clinical Microbiology Laboratory of the University of Virginia Health System (UVaHS), which serves a 619-bed tertiary care hospital, outpatient clinics in central Virginia, and since August 2010, a 40-bed long-term acute care hospital (LTACH). Weekly surveillance by perirectal swab was performed on all inpatients in units where there was a patient who was known to be colonized or infected with carbapenemase-producing Enterobacteriaceae (CPE), as previously described (32–34). Enterobacteriaceae from nonsurveillance clinical samples that were flagged as possibly producing an extended-spectrum β-lactamase (ESBL) or that had an ertapenem MIC of ≥1 μg/ml by automated susceptibility profiling (Vitek 2; bioMérieux, Durham, NC) underwent carbapenemase phenotypic testing using the modified Hodge test (August 2007 to June 2008) or the indirect carbapenemase test (July 2008 to September 2012) (35). Isolates identified as K. pneumoniae by the Vitek 2 system with a positive carbapenemase phenotypic test and/or meropenem or imipenem MIC of ≥1 μg/ml underwent *bla*_KPC_ PCR analysis, as previously described (4, 35). The first available KPC-*Kp* isolate for the index patient (30) and 36 out of 64 additional patients throughout the time period were selected for WGS at random by a laboratory technician in a blind manner (unaware of the clinical data). For a smaller cohort of individuals in whom longitudinal colonization with a KPC-*Kp* of the same sequence type (ST) was observed, the last isolate cultured from the patient was sequenced in addition to the first isolate in order to determine a molecular clock rate for the study K. pneumoniae. A subset of all the selected isolates had previously undergone pulsed-field gel electrophoresis (PFGE) and multilocus sequence typing (MLST), and those results have been previously reported (36).

### Clinical epidemiology and case definitions.

Infection control measures to limit the nosocomial transmission of CPE were implemented in our institution in 2007 although methods changed over time with new recommendations (31). Patients were prospectively identified, and the date, location in the hospital, and anatomical site of the relevant sample were recorded. Their detailed clinical characteristics were gathered retrospectively from electronic medical records. The initial culture with a CPE by phenotypic testing was considered the epidemiological acquisition event of *bla*_KPC_ by a patient. CPE-positive patients were considered at risk for transmitting CPE (including KPC-*Kp*) at any point during hospitalization, including prior to positive CPE culture and were considered to be carriers for the duration of the study. KPC-*Kp* isolates were classified as “imported” for patients without prior admission to UVaHS Medical Center/LTACH (UVaMC) and with a carbapenem-resistant K. pneumoniae culture before or within 48 h of transfer to UVaMC. For the remaining patients, the risk of acquiring KPC-*Kp* at UVaMC was based on any hospitalization at UVaMC in the previous 90 days and classified as “high” if there was a CPE carrier patient in the same unit simultaneously prior to isolation of a new KPC-*Kp* and “indeterminate” if the patient was admitted to UVaMC for ≥48 h but was never in the same hospital unit at the same time as a CPE carrier. The study was approved by the University of Virginia Institutional Review Board (protocol 13558).

### Whole-genome sequencing. (i) Host strain DNA preparation, sequencing, and sequence assembly.

DNA was extracted using a commercial kit (QuickGene DNA tissue kit S, Fujifilm, Japan) as previously described (37), and sequenced on the Illumina HiSeq 2000 instrument (for details, see supplemental material with accession numbers in Table S4 in the supplemental material). Reads were mapped against the reference K. pneumoniae sequence MGH78578 (RefSeq accession no. NC_009653) using Stampy (38). *De novo* assembly was performed using Velvet with automated optimization of assembly parameters using VelvetOptimiser as previously described (37). For the KPC-*Kp* and KPC-*Ko* isolates cultured from the index case (CAV1016 and CAV1015, respectively), single-molecule real-time sequencing using the Pacbio RSII instrument for full-genome and plasmid assembly was also undertaken to confirm plasmid structure with long-read methods (details in supplemental material) (39).

*In silico* MLST was performed for all isolates using the MLST scheme developed at Institut Pasteur (details in supplemental material) (40). Phylogenetic relationships between all of the KPC-*Kp* strains on the basis of single-nucleotide variant (SNV) differences distributed over the core mapped genome with respect to the reference K. pneumoniae genome were represented as a maximum likelihood tree (details in supplemental material). For two K. pneumoniae lineages, ST258 and ST941, within-ST comparisons of evolutionary relationships were made using ClonalFrame (41) to account for the impact of recombination.

The molecular clock was determined by comparing the sequence variation between the first and last isolate taken from each patient in the smaller cohort of individuals in whom longitudinal colonization had been observed. The clock was calculated using the time interval between the two samplings and applying a Bayesian model to estimate the evolutionary rate as described previously (24). This was then used to scale the ClonalFrame trees to estimate dating and dating intervals around the time to the most recent common ancestors (TMRCA) in the trees.

### (ii) Plasmid conjugation, recovery, identification, assembly, and analysis.

Plasmid transformants for the index case isolates (CAV1016 and CAV1015) were generated by extraction of plasmid DNA and subsequent electroporation into E. coli GeneHogs (Invitrogen, Grand Island, NY). DNA extraction and Illumina sequencing for transformants were undertaken as for host strains as described above.

Conjugation experiments of index case *bla*_KPC_ plasmids were performed via mating of E. coli J53 Rif^r^ strain to the clinical isolates CAV1016 and CAV1015 as well as the GeneHogs transformants, as previously described (42).

To recover plasmid sequence reads, all GeneHogs transformant reads were mapped using Stampy against reference sequence E. coli DH10B (RefSeq accession no. NC_010473.1). Unmapped reads of transformants representing putative plasmid sequences were then *de novo* assembled using A5 (43), and with additional gap closure, single closed plasmid structures were obtained for each transformant (pKPC_UVA01 and pKPC_UVA02; details in supplemental material).

The Tn*4401* sequence present in each clinical isolate was ascertained by BLASTn comparisons of *de novo* assemblies with the reference Tn*4401*b isoform (GenBank accession no. EU176013.1). Matching sequences were extracted, aligned, and compared to previously described isoform structures (Tn*4401*a to Tn*4401*e) and evaluated for the presence of deletions consistent with these characterized isoforms (44). Incompatibility group typing of pKPC_UVA01 and pKPC_UVA02 was done through *in silico* analysis by BLASTn comparisons with previously described incompatibility groups (45).

For each clinical isolate, reads were mapped using Burrows-Wheeler Aligner (BWA) version 0.7.5a against pKPC_UVA01 and pKPC_UVA02 (details in supplemental material). Plasmids were considered to be present in a clinical isolate if at least 90% of the reference sequence had coverage of ≥10.

Long-range PCR was performed on all 37 initial KPC-*Kp* isolates in a blind manner by a researcher who was unaware of WGS results to evaluate the presence of Tn*4401* in the *bla*_KPC_ plasmids from the CAV1016 and CAV1015 isolates. For both pKPC_UVA01 and pKPC_UVA02, primers were designed to amplify two overlapping fragments, together spanning Tn*4401*. For each fragment, one primer was located in *bla*_KPC_, and the other was located in a region of the plasmid adjacent to Tn*4401*. Amplicons were between 4,041 and 11,841 bp and were all sequenced to confirm content with detailed methods contained in the supplemental material (see Table S1 in the supplemental material).

### Nucleotide sequence accession numbers.

The sequences of the new plasmids described in this study have been deposited in GenBank under accession numbers CP009465 and CP009466.

## RESULTS

### Clinical and epidemiological characteristics.

Of the 250 patients with suspected CPE by phenotypic evaluation, 226 patients had evaluable isolates collected. Of these 226 patients, 202 (89.3%) had at least one *bla*_KPC_ PCR-positive Enterobacteriaceae, including 64 (28.3%) patients with at least one *bla*_KPC_ PCR-positive K. pneumoniae isolate. Initial KPC-*Kp* isolates from 37 patients underwent WGS, of which 23 (62%) were sourced from clinical specimens derived from urine samples (10 isolates), respiratory samples (6 isolates), abdominal wound samples (4 isolates), and blood samples (3 isolates). Fourteen (38%) isolates were cultured from perirectal surveillance specimens. Of these patients, eight (57%) later yielded a clinical specimen with *bla*_KPC_-positive Enterobacteriaceae.

Seven patients imported KPC-*Kp* into UVaMC as follows: (i) the index case (*n* = 1) (30); (ii) patients with carbapenem-resistant K. pneumoniae-positive culture obtained at an outside hospital prior to transfer (*n* = 3); (iii) patients with a KPC-*Kp*-positive urine culture obtained as an outpatient (*n* = 2); and (iv) a patient with a KPC-*Kp*-positive culture within 48 h of admission (*n* = 1). Of the 30 patients at risk of acquiring KPC-*Kp* in UVaMC, the median duration of hospitalization in the 90 days prior to isolation of CPE was 20 days (range, 2 to 81 days). Eighty-three percent (25/30) were considered high risk for acquisition at UVaMC and 17% (5/30) indeterminate risk.

### Host strain diversity, risk of acquisition, and evolutionary clock.

**FIG 1:**
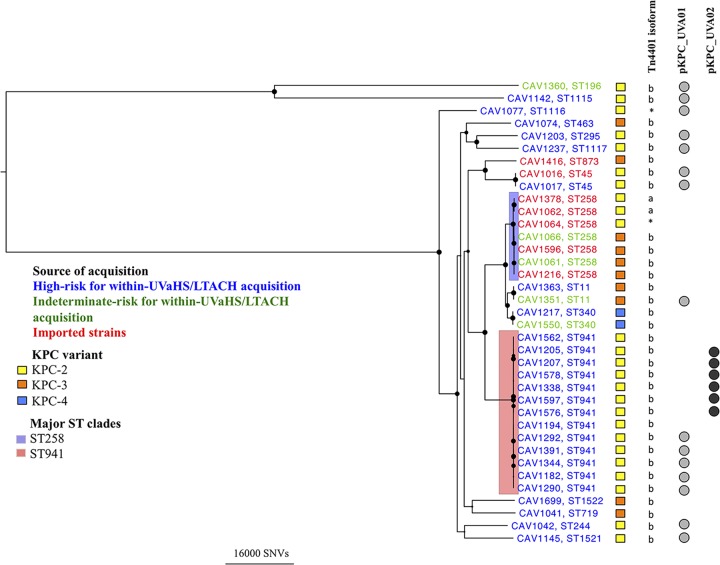
Maximum likelihood phylogeny of core study Klebsiella pneumoniae carbapenemase (KPC)-producing K. pneumoniae sequences, in association with *bla*_KPC_ allele, characterized Tn*4401* isoforms, *bla*_KPC_ plasmids (pKPC_UVA01 and pKPC_UVA02), and risk of within-hospital acquisition. Black circles at the nodes represent bootstrap values of >70% with the size of the circle reflecting the degree of support. The largest circles have bootstrap values of 100%. Tn*4401* isoforms (a to e) and unclassified Tn*4401* isoforms (*) are shown. Abbreviations: ST, sequence type; UVaHS/LTACH, University of Virginia Health System/long-term acute care hospital; SNVs, single-nucleotide variants.

Sixteen distinct STs were identified, two of which were novel, from the 37 K. pneumoniae isolates (Fig. 1; see Table S2 in the supplemental material). For the 11 and 13 isolates that had previously undergone traditional MLST and PFGE, respectively, the *in silico* MLST results matched the prior results, and the diversity seen on PFGE was also congruent with the WGS phylogeny (4, 36). Forty-six percent (17/37) of the isolates belonged to discrete STs with two or fewer isolates per ST. There were two STs represented by more than two isolates: ST941 (13/37 [35%]), which has not previously been described in association with *bla*_KPC_, and the epidemic ST258 (7/37 [19%]). None of the patients with ST258 KPC-*Kp* isolates were considered high risk for acquisition within UVaMC; five of the isolates were imported, and two patients had indeterminate risk (Fig. 1). All of the patients with ST941 were considered at high risk for acquisition within UVaMC. Excluding the indeterminate acquisition cases, patients with ST258 were more likely to acquire KPC-*Kp* outside UVaMC than non-ST258 (5/5 versus 2/26; *P* = 0.0001 by Fisher's exact test).

**FIG 2:**
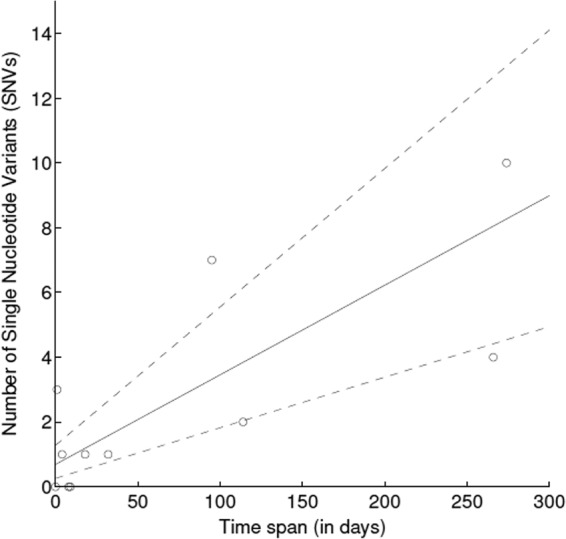
Molecular clock estimate calculated using Bayesian inference on genetic data from the first and last Klebsiella pneumoniae isolates producing K. pneumoniae carbapenemase sampled from study individuals. The mean molecular clock estimate (solid line) and 95% credibility interval (dashed lines) are shown.

Data from 11 individuals with paired longitudinal samples sharing the same ST were appropriate for the assessment of the molecular clock, with a median of 18 days (range, 0 to 274 days) and 1 SNV (range, 0 to 10 SNVs) between paired samples. The molecular clock was calculated as being 1.9 × 10^−6^ substitutions/called site/year (95% credibility interval [95% CI], 1.1 × 10^−6^ to 2.9 × 10^−6^), equating to 10.1 substitutions/genome/year (95% CI, 5.7 to 15.6) (Fig. 2).

**FIG 3:**
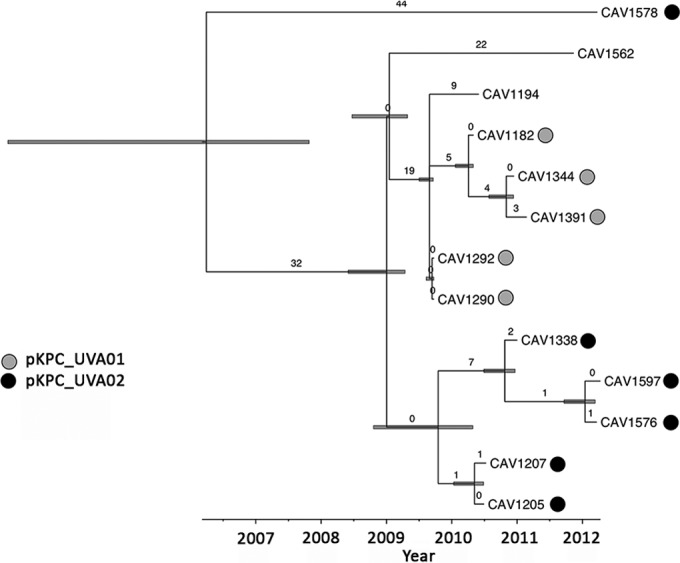
Time-scaled representation of genetic relationships between multilocus sequence type ST941 strains, in association with predicted Klebsiella pneumoniae carbapenemase plasmids (pKPC_UVA01 and pKPC_UVA02). The numbers of mutational substitutions are represented numerically on the branches, with bars at nodes indicating 95% credibility intervals around the estimates of the time to the most recent common ancestors.

Based on a ClonalFrame host strain analysis of the ST941 isolates, the TMRCA dated to mid-2006 with the 95% credibility interval ranging from 2003 to 2008 (Fig. 3). This includes the time of first appearance in 2007 of *bla*_KPC_ in UVaMC and would be consistent with this lineage acquiring *bla*_KPC_ soon after its appearance in the local hospital ecosystem.

**FIG 4:**
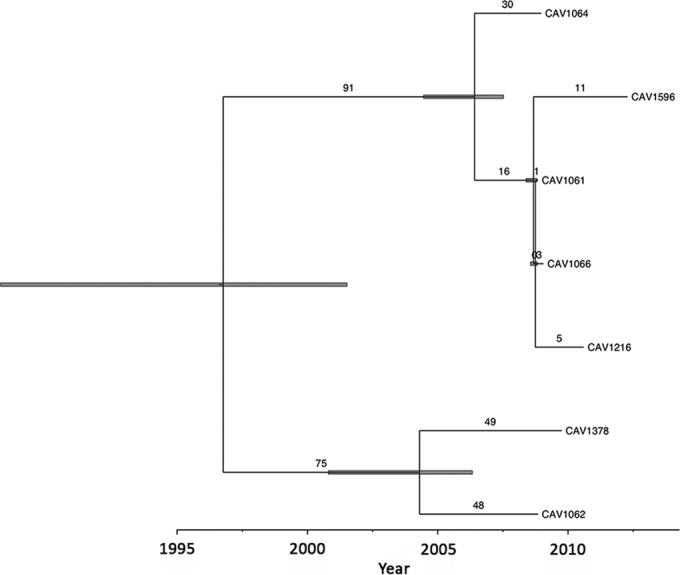
Time-scaled representation of genetic relationships between multilocus sequence type ST258 strains. The number of mutational substitutions are represented numerically on the branches, with gray bars at nodes indicating the uncertainty of 95% credibility intervals around the estimates of the time to the most recent common ancestors.

A ClonalFrame analysis of the seven ST258 isolates demonstrated the TMRCA dating to 1997 (range, 1988 to 2002; Fig. 4). Four of these isolates (CAV1596, CAV1061, CAV1066, and CAV1216) were closely related (≤15 SNVs) and shared proximity to a single outside hospital. The remaining three ST258 KPC-*Kp* were genetically divergent and imported to UVaMC from three separate outside hospitals in the mid-Atlantic region of the United States. There was no epidemiological evidence of sustained ST258 transmission within UVaMC.

### Plasmids, relationships to host KPC-*Kp*, and spread of *bla*_KPC_.

**FIG 5:**
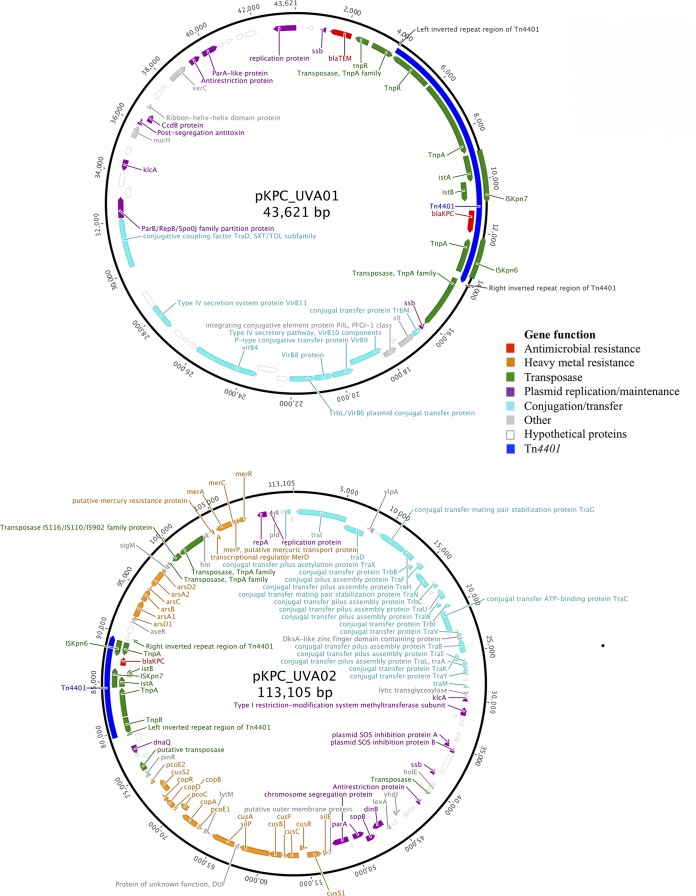
Annotated novel *bla*_KPC_ plasmids pKPC_UVA01 and pKPC_UVA02 from Klebsiella pneumoniae and Klebsiella oxytoca isolated from the index patient.

We previously described an index case with KPC-*Kp* (CAV1016) who was admitted to the UVaMC with subsequent *bla*_KPC_ plasmid dispersal to other Enterobacteriaceae (4). *De novo* assembly of the index *bla*_KPC_ plasmid from CAV1016 was generated from the E. coli GeneHogs transformant (GH1016). It produced a 43,621-bp, closed, nontypeable (by incompatibility group) Tn*4401*-containing plasmid (pKPC_UVA01) with little homology to any previously described plasmids (GenBank accession no. CP009465) (Fig. 5).

The index case also harbored a KPC-*Ko* (CAV1015) (4), from which a second GeneHogs transformant (GH1015) was generated. Assembly of plasmid reads from this transformant yielded a 113,105-bp, closed, nontypeable plasmid structure containing Tn*4401* and designated pKPC_UVA02 (GenBank accession no. CP009466) (Fig. 5).

To further confirm the sequence and structure of these two *bla*_KPC_ plasmids, we performed long-read PacBio sequencing of the corresponding clinical strains, CAV1015 and CAV1016. The resulting assemblies yielded *bla*_KPC_ plasmid sequences that were identical to those generated from the transformants.

CAV1015 and CAV1016 and plasmid transformants GH1015 and GH1016 were successfully mated to E. coli J53 Rif^r^ and confirmed by *bla*_KPC_ PCR, thus demonstrating that both pKPC_UVA01 and pKPC_UVA02 are freely conjugative plasmids.

To ascertain the presence of the two *bla*_KPC_ plasmids in the 37 KPC-*Kp* isolates, Illumina reads from each clinical isolate were mapped against the two novel plasmid references. This demonstrated the presence of pKPC_UVA01 in 41% (15/37) of the KPC-*Kp* isolates, spanning 10 STs over a 3.5-year period (Fig. 1; see Table S2 in the supplemental material). Likewise, pKPC_UVA02 was found in 16% (6/37) of isolates over nearly 2 years, and all of these isolates belonged to ST941.

To demonstrate that Tn*4401* was present in the predicted locations in pKPC_UVA01 and pKPC_UVA02 structures, long-range PCR was performed on all clinical isolates using primers spanning the Tn*4401* insertion sites. PCR products of the correct sizes were obtained for all isolates classified as containing pKPC_UVA01 or pKPC_UVA02 (Fig. 1), and the sequences were confirmed to correspond to the expected amplicons (see Table S3 in the supplemental material).

Time-scaled, phylogenetic analysis of the 13 ST941 isolates revealed that they had most likely independently acquired pKPC_UVA01 and pKPC_UVA02 at different points in time within UVaMC (Fig. 3). Five isolates contained pKPC_UVA01, and these isolates formed a cluster together with one additional isolate (CAV1194), with a common ancestor in 2009. The sixth isolate of this cluster (CAV1194) was considered to have an unresolved *bla*_KPC_ plasmid. Another discrete ST941 cluster of five isolates, with a common ancestor in 2009, contained pKPC_UVA02. A single more divergent isolate also containing pKPC_UVA02 shared a common ancestor with the remaining isolates dating to mid-2006. Ten isolates, all part of the two discrete clusters of five isolates, each differed by ≤3 SNVs from at least one other isolate, consistent with recent shared acquisition from a common source which could include person-to-person transmission (20).

### *bla*_KPC_ and Tn*4401* variants and their relationship to host plasmids.

Sixty-eight percent of isolates (25/37) contained *bla*_KPC-2_, 27% (10/37) contained *bla*_KPC-3_ and 5% (2/37) contained *bla*_KPC-4_ (Fig. 1). pKPC_UVA01 contained a *bla*_KPC-2_ allele and the Tn*4401*b isoform, with two exceptions: a single *bla*_KPC-3_ in CAV1351 and a Tn*4401* isoform from CAV1077 which was not identical in structure to any of the previously described Tn*4401*a to Tn*4401*e isoforms. This could occur as a result of alterations in *bla*_KPC_ and/or Tn*4401* over time within the pKPC_UVA01 structure or repeated insertion of different variants into the same site in this plasmid. All five KPC-*Kp* isolates carrying pKPC_UVA02 had the *bla*_KPC-2_ allele and Tn*4401*b isoform.

## DISCUSSION

This WGS-based analysis of KPC-*Kp* dating from August 2007 when *bla*_KPC_ was likely introduced into and became endemic in a single U.S. hospital has demonstrated the following: (i) high genetic diversity of KPC-*Kp* lineages, with 16 different STs identified among 37 isolates; (ii) dispersal of two newly described conjugative KPC plasmids, pKPC_UVA01 and pKPC_UVA02, among K. pneumoniae lineages, with 10 different STs containing pKPC_UVA01; (iii) emergence of a new KPC-*Kp* lineage ST941 which has acquired these two plasmids (and possibly others) likely at different time points and then undergone local spread; and (iv) multiple independent importation events of the epidemic KPC-*Kp* ST258 lineage, which has not spread or become established in the local hospital ecosystem.

These observations build on the study reported in 2011 suggesting that there was dissemination of a single plasmid among different genera in UVaMC, but in which only three K. pneumoniae isolates were investigated, and WGS was not performed, thus preventing fine-scale resolution of phylogenetic relationships (4).

The wide diversity of lineages of K. pneumoniae encountered in UVaMC is at variance with the widely held view that the spread of KPC-*Kp* is most often restricted to ST258 K. pneumoniae or highly related strains in the same clonal complex (2, 5, 46) and contrasts with the majority of descriptions of KPC-*Kp* nosocomial dissemination (8, 20, 47–49). It is not clear why KPC-*Kp* ST258 was not involved in ongoing transmission as seen elsewhere, despite multiple introductions into the institution. Similarly, the reason for the differences in transmission epidemiology between ST258 and ST941 is not evident, but it may be related to the initial introduction, promiscuity, and/or selective advantage afforded by resistance plasmids rather than a single clonal complex-plasmid combination.

Our data support the hypothesis that local dispersal of pKPC_UVA01 and pKPC_UVA02 among diverse K. pneumoniae bacteria has most likely occurred as a result of intra- or interspecies conjugative transfer from other K. pneumoniae bacteria or various species of Enterobacteriaceae at multiple time points within the hospital ecosystem (4, 30). A similar experience was observed recently in Spain and Norway where multiple KPC-positive strains and plasmids were identified in a single clinical setting, also giving rise to a complex molecular epidemiological picture (18, 19). However, these studies used lower-resolution typing of isolates, limiting the analysis of relationships between host bacteria and their *bla*_KPC_ plasmids, and were unable to establish timelines for plasmid introduction and strain dissemination as shown here. In these situations, the extent of plasmid spread by direct patient-to-patient transmission, through other human intermediaries, or through exchange in environmental reservoirs, remains to be elucidated and requires analysis of additional environmental samples, strains carried by patients, and other Enterobacteriaceae species.

There are several limitations of this study. One obvious shortcoming was that we chose to analyze only a relatively small subset of the most globally relevant species of CPE, namely, K. pneumoniae. In spite of this single-species approach, which has undoubtedly left gaps in the total plasmid/transposon/resistance gene epidemiology, a convoluted dynamic relationship of plasmid and strain interaction is clearly observed. Where resistance has become endemic and involves many genera of bacteria, it would be unsurprising were an even more complicated picture observed than revealed in this study. Additionally, due to the repetitive nature of resistance plasmids, it is often impossible to fully assemble them from short-read sequencing data, and it can be difficult to confirm the contiguity of the structure around variable regions such as the Tn*4401* transposon. As a result of this issue, 43% (16/37) of the isolates have uncharacterized *bla*_KPC_ plasmid structures which will require additional experimentation to resolve.

In summary, we have used WGS analysis to identify two novel *bla*_KPC_ plasmids likely introduced by an index patient in 2007. We then tracked both plasmids through multiple KPC-*Kp* strains using short-read mapping combined with long-range PCR to confirm the colocation of *bla*_KPC_ within the newly described plasmids. This yields insights into the complex dynamics of plasmid and strain interactions among KPC-*Kp* as *bla*_KPC_ becomes endemic at a single institution over a period of 5 years. This dynamic includes introduction of novel, emergent plasmids into a KPC-*Kp* lineage (ST941) undergoing local clonal expansion and sustained transmission, amid a backdrop of local plasmid spread to other lineages and importation of the nationally epidemic ST258 KPC-*Kp* strains as discrete events. Although knowledge gaps remain surrounding extension to other species and potential plasmid reservoirs involved in the spread of resistance, the high-resolution, joint analysis of plasmid and host bacterial diversity provides new insights into the paths by which KPC-*Kp* has become endemic in a hospital over time.

## Supplementary Material

Supplemental material
