# Supplementary material for: Klebsiella pneumoniae Carbapenemase (KPC)-Producing K. pneumoniae at a Single Institution: Insights into Endemicity from Whole-Genome Sequencing
Source: Antimicrob Agents Chemother. 2015 Feb 11;59(3):1656–63. doi: 10.1128/AAC.04292-14 (PMC4325807; doi:10.1128/AAC.04292-14)
Supplement: Supplemental material [file supp_59_3_1656__index.html]

Supplemental material 

# Klebsiella pneumoniae Carbapenemase (KPC)-Producing K. pneumoniae at a Single Institution: Insights into Endemicity from Whole-Genome Sequencing

## Supplemental material

**Files in this Data Supplement:**

- Supplemental file 1 -

  Additional experimental details and Supplemental Tables S1 to S5.

  PDF, 453K
