# Supplementary material for: Klebsiella pneumoniae Carbapenemase (KPC)-Producing K. pneumoniae at a Single Institution: Insights into Endemicity from Whole-Genome Sequencing
Source: Antimicrob Agents Chemother. 2015 Feb 11;59(3):1656–63. doi: 10.1128/AAC.04292-14 (PMC4325807; doi:10.1128/AAC.04292-14)
Supplement: Supplemental material [file AAC.04292-14_zac003153789so1.pdf]

Supplementary information

Illumina sequencing

DNA was sequenced at the Wellcome Trust Centre for Human Genetics (WTCHG), Oxford, UK, using the Illumina HiSeq 2000 platform. The study DNA extracts were sequenced as part of larger sequencing datasets batched as 96 samples, which included sequencing of the reference MGH78578 *K. pneumoniae* strain as a sequencing control. A combination of standard Illumina and in-house protocols were used to produce multiplexed paired-end libraries. Reads were 2 x 150 bp, with an average of 2,738,876 reads per sample across all *K. pneumoniae* study isolates, giving rise to approximately 77x coverage per sample with a median insert size of 442.

Reference-based mapping for the host *K. pneumoniae* bacterial strain analysis

For reference-based mapping to *K. pneumoniae* MGH78578, reads were mapped using Stampy v1.0.21 (without Burrows-Wheeler Aligner pre-mapping, and an expected substitution rate of 0.01).

Repetitive regions of the reference genomes were identified using BLAST, and excluded from base calling. Single nucleotide variants (SNVs) were determined across all mapped non-repetitive sites using SAMtools (version 0.1.18) mpileup with the extended base-alignment quality flag, after parameter tuning based on bacterial sequences (options '-E -M0 –Q25 -q30 -m2 -D –S' and otherwise default values). GATK VariantAnnotator (version 1.4.21) was used to create variant call format (VCF) files of annotated variant sites. We only called sites that were supported by ≥5 reads, including one in each direction. A consensus of ≥0.90 of high

quality bases was also required to support a call. Calls required the ratio of high quality bases to total reads spanning the site of interest to be ≥0.35, and the root-mean-square mapping quality of covering reads to be ≥30. SNVs were only called if they had quality ≥25 and were homozygous under a diploid model. For sites where one or more of these criteria failed, no base was called (i.e. the site was treated as 'N').

Core SNVs (defined as the set of SNVs amongst sequences relative to the reference and excluding sites where any isolates had 'N' or '-' calls) were then identified for the three phylogenetic datasets: the whole group of sequences (37 strains), the ST941 group (13 sequences), and the ST258 group (seven sequences).

Filtered SNVs for each isolate were then reinserted into the reference genome MGH78578 sequence, resulting in an alignment of 37 modified reference sequences for comparisons across the whole dataset, 13 sequences for the ST941 group and seven sequences for the ST258 group.

Whole genome *de novo* assemblies

Reads were *de novo* assembled with Velvet version 1.0.18, with automated optimization of assembly parameters (K, -exp_cov, -cov_cutoff) to achieve the highest contig N50 value and largest number of bases in contigs >1 kilobase - this was done using VelvetOptimiser version 2.1.7 with a kmer range of 75-149, and resulted in median kmer-length, expected coverage and coverage cut-offs for the dataset of 83, 21.5 and 6.13 respectively. The median number of contigs for the assemblies was 229 (range: 110-1021); the same values for contigs >1

kilobase were 111 (range: 40-169), and for the contig N50 metric, 184,625 (range: 136,106-474,028).

Long Read Sequencing and Assembly

For the index KPC-Kp and KPC-Ko isolates we performed single molecule real-time sequencing using the Pacbio RSII for full genome and plasmid assembly. DNA library preparation and sequencing were performed according to the manufacturer's instructions and reflects the P5-C3 sequencing enzyme and chemistry, respectively. Briefly, after library preparation and Blue-Pippin size selection using a 7kbp to 50kbp selection, the polymerase-template complex was bound to the P5 enzyme using a ratio of 10:1 polymerase to SMRTbell at 0.5 nM for 4 hours at 30ºC and then held at 4ºC until ready for magbead loading, prior to sequencing. The magbead-loaded, polymerase-bound, SMRTbell libraries were placed onto the RSII machine at a sequencing concentration of 125 pM and configured for a 240-minute continuous sequencing run to allow for the largest sub-read length possible for the most contiguous assembly. Sequencing was conducted across 2 SMRTcells for CAV1015 and a single SMRTcell for CAV1016, resulting in read N50 values of 10,800 bp and 10,600 bp, respectively. The PacBio de novo assemblies were conducted using HGAP3 version 2.2.0 software, resulting in phased chromosomal and plasmid contigs.

Phylogenetic analysis of the relationships of host strains

A maximum-likelihood tree of the complete dataset was constructed using RaxML version 7.7.6, implementing: (i) a generalized time-reversible nucleotide substitution model, (ii) fixing the proportion of invariant sites at 0, (iii) allowing for four relative substitution rate

categories, and (iv) using the rapid bootstrapping algorithm outputting the best ML tree with 100 replicates. An example command line was:

```
/RAxML-7.7.6/raxmlHPC-PTHREADS-SSE3 -T 5 -f a -s
input_sequence_alignment_file.phy  -m GTRGAMMA -p 12345 -c 4 -x 12345 -# 100 -n
kpc_uva_all_snpytree_08092014_raxml_rapid_bootstrap
```

For within-lineage phylogenetic analysis, ClonalFrame version 1.2 was used in order to account for any recombination, particularly segments which may have been imported from more divergent lineages. Three separate runs were performed for both the ST941 and the ST-258 clusters, on the alignments of modified reference strains (with SNVs reinserted), as described above. The ClonalFrame settings were as follows:

- Number of MCMC iterations following the burn-in period = 2000
- Number of burn-in iterations = 2000
- Number of iterations between recording parameter values in the posterior sample (the thinning interval) = 2

The basic command used for this analysis was:

```
./ClonalFrame -x 2000 -y 2000 -z 2 input_file > output_file
```

Convergence of the runs was assessed by comparing the similarity of the run outputs, with reference to the global parameters (theta, rho, nu, and delta) and the phylogeny. In addition, the Gelman & Rubin test statistic was calculated for the global parameters for pairs of runs. Consensus trees, based on the union of posterior samples for each of the triplicate datasets, were determined. Time-scaling was done using the mutation rate calculated from the longitudinally sampled paired KPC-*Kp* study isolates.

In silico multi-locus sequence typing (MLST) and incompatibility group typing for sequenced isolates

A BLAST database of the alleles for the MLST loci (*rpoB*, *gapA*, *mdh*, *pgi*, *phoE*, *infB*, *tonB*) and STs as defined in the scheme developed at Institut Pasteur (http://www.pasteur.fr/recherche/genopole/PF8/mlst/Kpneumoniae.html) was established, and sequence matches to *de novo* assembled contigs were identified using BLASTn version 2.2.23 (default settings, except the e-value, which was set at 0.01) with an in-house python script to process and format results. Any 100% match to an allelic sequence in the database was taken as the allele for that locus for that isolate. Partial matches were obtained for *gapA* (two isolates), *phoE* (1 isolate) *tonB* (8 isolates), *rpoB* (2 isolates), and *mdh* + *tonB* (one isolate). BLAST was repeated using default settings except for a word size of 17 and an e-value of 0.001; in addition, any partial matches spanning several contigs sharing an overlapping alignment were joined together to give a single sequence. Once all seven allelic sequences were determined, an ST was assigned, as per the Pasteur database.

For the three cases where uncertainty remained in the results from both BLAST processes, or the two cases where there were inconsistencies between the two BLAST methods, the filtered calls in the VCF file derived from the reference-mapped data (at the relevant MLST locus position with respect to the reference) were interrogated, and an allele was determined on that basis. In the first three cases, visual inspection of the VCF files showed support for the allelic variant that was called with a minimum of 15 high quality reads supporting base calls across the locus (CAV1290, CAV1016, CAV1017). In the second two cases, mapping data supported the insertion of short sequences consistent with the results of the first BLAST

approach; these allelic calls were also supported by conventional MLST typing using PCR and subsequent Sanger sequencing.

Typing of plasmid incompatibility (Inc) groups was similarly carried out using a BLAST-based approach to probe *de novo* assembled contigs for the transformant plasmids, having established a database of reference Inc group sequences.

Mapping and processing of transformant strains

Sequenced plasmid transformants were mapped to *E. coli* DH10B using Stampy as described above. Unmapped reads (corresponding to presumed plasmid sequences) were extracted from the mapped .bam files using features available in Samtools and Picard (version 1.66), and the following commands:

```
samtools view -b -f 12 input_filename > output_filename
```

```
java -jar ./picard/v1.66/SamToFastq.jar input_filename=$1 fastq=$2 second_end_fastq=$3
```

Unmapped, paired end reads represented in the resulting fastq files were then assembled with A5, using the following command:

```
./a5_pipeline.pl fastq second_end_fastq ouput_prefix
```

This resulted in one and two scaffolds respectively for the electroporated transformants corresponding to the index patient's *K. pneumoniae* and *K. oxytoca*. Comparisons with the

velvet assemblies derived from the complete sets of reads for each transformant were used to close gaps between contigs. Mapping of the complete sets of reads to the resulting plasmid sequences was used to confirm plasmid structure and correct minor sequence errors in the assemblies. This resulted in a single, closed plasmid structure for each transformant, GH1016 (pKPC_UVA01) and GH1015 (pKPC_UVA02).

Plasmid analysis

For plasmid analysis, reads were mapped to pKPC_UVA01 and pKPC_UVA02 using BWA mem, version 0.7.5a, with default parameters, and the depth of coverage for each position on the reference was ascertained with samtools mpileup version 0.1.19 with the -A flag and otherwise default parameters. An absolute value of 10 reads was used as a cutoff for presence at each position. Custom python scripts were used to calculate the proportion of positions with read depth above this threshold. These values are presented as % plasmid presence in Supplementary table 2. We defined a cutoff of 90% of a plasmid reference with read depth ≥ 10 as a measure of the presence of a plasmid as a whole; this definition was used to define presence / absence as depicted in Figure 1. The raw precise values (% of reference with read depth above threshold) are indicated in Supplementary table 2.

Long Range PCR for pKPC_UVA01 and pKPC_UVA02

Polymerase chain reaction (PCR) was performed using LongAmp™ *Taq* DNA polymerase (New England Biolabs, USA). 1 µL sample DNA was combined with 5 µL LongAmp™ *Taq* DNA buffer, 0.75 µL of 10 mM dNTPs, 0.5 µL of 10 pM forward and reverse primer (Supplementary table 1), 1 µL of LongAmp™ *Taq* DNA polymerase, and 16.25 µL nuclease-

free H_2O to a final reaction volume of 25 µL. PCR reactions were performed under the following conditions: 94°C for 30s; 30 cycles of 94°C for 15s and 65°C for 6 minutes (amplicon lengths up to 7 kb, performed for region 1 of pKPC_UVA01 and pKPC_UVA02) or 11 minutes (amplicon lengths up to 13 kb, performed for region 2 of pKPC_UVA01 and pKPC_UVA02); final extension at 65°C for 10 minutes.

All four PCR reactions were performed on all 37 study samples, including CAV1016, the index patient *Klebsiella pneumoniae* strain, which acted as a positive control for pKPC_UVA01, as well as CAV1015, the index patient *Klebsiella oxytoca*, which acted as a positive control for pKPC_UVA02. Reactions were performed using the 2720 thermal cycler (Applied Biosystems, USA); post-amplification samples were cleaned with 1.8x AMPure XP beads (Beckman Coulter, UK). The DNA bound to AMPure beads was washed twice with 70% ethanol according to the manufacturer’s instructions, and the DNA eluted in 30 µL 1x tris-ethylenediaminetetraacetic acid buffer. PCR products were visualised using 1% agarose gel electrophoresis. All samples generating amplicons at the predicted molecular weight were selected for sequencing (Supplementary table 3).

Samples containing amplicons were quantified using the Qubit 2.0 Fluorometer (LifeTechnologies, USA) and normalised to 0.2 ng/µL. Sequencing libraries were prepared using the Nextera XT library preparation kit (Illumina, USA, Part #15031942 rev. C, October 2012) following manufacturer’s instructions with the following modifications; the limited cycle PCR was extended from 12 to 15 cycles, and post-PCR normalisation was performed manually following quantitation using the Qubit and qualification using the 2200 TapeStation (Agilent Technologies, USA). Sequencing was performed using a MiSeq Reagent kit v2 2x150bp paired end read cartridge.

Illumina reads were subsampled to give 5000 randomly selected read pairs for each sequenced PCR product. Adaptor trimming was performed with cutadapt version 1.5, specifying the adaptor sequence as -a CTGTCTCTTATACACATCT. Trimmed reads were assembled using spades version 3.1.1 with a kmer of 99 and the --careful option enabled. Blastn was used to assess similarity between the resulting assembly and the expected PCR product.

Supplementary table 1: Primer pairs used for long range amplification of pKPC_UVA01 and pKPC_UVA02

Primer target	Primer name	Forward primer (5'-3')	Tm (°C)	Amplicon length (bp)
pKPC_UVA01 region 1	pUVA01_reg1_a_F	5'-GTGGTCTACCTGAAATTCTTTACGTCTTGC-3'	59.9	11841
	pUVA_kpc_a_R	5'-GTGTAGACGGCCAACACAATAGGTGC-3'	59.8	
pKPC_UVA01 region 2	pUVA_kpc_c_F	5'-GTCTAGTTCTGCTGTCTTGTC-3'	51.3	6454
	pUVA01_reg2_c_R	5'CTCACCACCTCGTTTCATTC-3'	50.7	
pKPC_UVA02 region 1	pUVA01_reg1_a_F	5'-AGGCTGCTGACTCTGTCACGTTC-3'	59.8	9038
	pUVA_kpc_a_R	5'-GTGTAGACGGCCAACACAATAGGTGC-3'	59.8	
pKPC_UVA02 region 2	pUVA_kpc_b_F	5'-ACGCGATGGATACCGGCTCAGG-3'	61.2	4041
	pUVA01_reg2_b_R	5'-CGATTGCTTCCGGGCAGCTAATTC-3'	60.8	

Supplementary table 2. Molecular and epidemiological summary of first *Klebsiella pneumoniae* carbapenemase-producing *K. pneumoniae* (KPC-*Kp*) isolates collected from 37 study patients

Isolate name	Date of Isolation	Risk of in-hospital acquisition	Multi locus Sequence Type	KPC allelle	Tn*4401* long range PCR result	Plasmid Mapping	
						pKPC_UVA01	pKPC_UVA02
CAV1016	Aug-07	Index case	45	2	pKPC_UVA01	100	20
CAV1017	Oct-07	High	45	2	pKPC_UVA01	100	20
CAV1042	Apr-08	High	244	2	pKPC_UVA01	100	30
CAV1041	Apr-08	High	719	3	Neither	30	29
CAV1061	Nov-08	Indeterminate	258	3	Neither	34	35
CAV1062	Nov-08	Imported	258	2	Neither	33	33
CAV1064	Dec-08	Imported	258	2	Neither	18	31
CAV1066	Jan-09	Indeterminate	258	3	Neither	35	37
CAV1077	Feb-09	High	1116	2	pKPC_UVA01	100	28
CAV1074	Mar-09	High	463	3	Neither	23	34
CAV1142	Aug-09	High	1115	2	pKPC_UVA01	100	25
CAV1145	Sep-09	High	1521	2	pKPC_UVA01	100	38

CAV1292	Sep-09	High	941	2	pKPC_UVA01	100	38
CAV1290	Sep-09	High	941	2	pKPC_UVA01	100	38
CAV1378	Sep-09	Imported	258	2	Neither	34	34
CAV1360	Nov-09	Indeterminate	196	2	pKPC_UVA01	100	31
CAV1363	Dec-09	High	11	3	Neither[a]	71	37
CAV1182	Apr-10	High	941	2	pKPC_UVA01	100	39
CAV1194	May-10	High	941	2	Neither	34	38
CAV1237	Jun-10	High	1117	2	pKPC_UVA01	100	30
CAV1203	Jun-10	High	295	2	pKPC_UVA01	100	15
CAV1205	Jun-10	High	941	2	pKPC_UVA02	34	100
CAV1207	Jul-10	High	941	2	pKPC_UVA02	34	100
CAV1217	Aug-10	High	340	4	Neither	25	43
CAV1216	Aug-10	Imported	258	3	Neither	34	34
CAV1344	Dec-10	High	941	2	pKPC_UVA01	100	37
CAV1338	Dec-10	High	941	2	pKPC_UVA02	34	100
CAV1351	Feb-11	Indeterminate	11	3	pKPC_UVA01	100	29
CAV1391	Feb-11	High	941	2	pKPC_UVA01	100	38
CAV1416	May-11	Imported	873	3	Neither	34	24
CAV1550	May-11	Indeterminate	340	4	Neither	28	33
CAV1562	Nov-11	High	941	2	Neither	34	38
CAV1576	Mar-12	High	941	2	pKPC_UVA02	34	100
CAV1597	Apr-12	High	941	2	pKPC_UVA02	34	100
CAV1596	Apr-12	Imported	258	3	Neither	34	15
CAV1699	Sep-12	High	1522	3	Neither	30	29
CAV1578	Mar-12	High	941	2	pKPC_UVA02	23	96

[a]Only contained one of two fragments for Tn*4401* pKPC_UVA01 insertion

Supplementary table 3. Sequencing results from long fragment PCR products.

Sample	Plasmid	Region	Expected amplicon length	Contigs	Total length	Longest contig	Best hit length	Best blastn hit identity (%)
CAV1017	pKPC_UVA01	1	11841	1	11826	11826	11826	100
CAV1042	pKPC_UVA01	1	11841	1	11827	11827	11827	100
CAV1077	pKPC_UVA01	1	11841	1	11639	11639	10763	100
CAV1142	pKPC_UVA01	1	11841	1	11869	11869	11834	100
CAV1145	pKPC_UVA01	1	11841	1	11826	11826	11826	100
CAV1182	pKPC_UVA01	1	11841	1	11827	11827	11827	100
CAV1203	pKPC_UVA01	1	11841	1	11827	11827	11827	100
CAV1237	pKPC_UVA01	1	11841	1	11827	11827	11827	100
CAV1290	pKPC_UVA01	1	11841	1	11827	11827	11827	100
CAV1292	pKPC_UVA01	1	11841	1	11842	11842	11834	99.98
CAV1344	pKPC_UVA01	1	11841	2	14124	11826	11826	100
CAV1351	pKPC_UVA01	1	11841	1	11826	11826	11826	100
CAV1360	pKPC_UVA01	1	11841	1	11828	11828	11828	100
CAV1363[a]	pKPC_UVA01	1	11841	1	11826	11826	11826	100
CAV1391	pKPC_UVA01	1	11841	1	11857	11857	11826	100
CAV1016	pKPC_UVA01	1	11841	1	11826	11826	11826	100
CAV1017	pKPC_UVA01	2	6454	1	6443	6443	6443	100
CAV1042	pKPC_UVA01	2	6454	1	6457	6457	6440	100
CAV1077	pKPC_UVA01	2	6454	1	6442	6442	6442	100
CAV1142	pKPC_UVA01	2	6454	1	6439	6439	6439	99.98
CAV1145	pKPC_UVA01	2	6454	1	6443	6443	6443	100
CAV1182	pKPC_UVA01	2	6454	1	6440	6440	6440	100

CAV1203	pKPC_UVA01	2	6454	1	6443	6443	6443	100
CAV1237	pKPC_UVA01	2	6454	1	6443	6443	6443	100
CAV1290	pKPC_UVA01	2	6454	1	6443	6443	6443	100
CAV1292	pKPC_UVA01	2	6454	1	6470	6470	6447	99.98
CAV1344	pKPC_UVA01	2	6454	1	6440	6440	6440	99.98
CAV1351	pKPC_UVA01	2	6454	1	6443	6443	6443	99.98
CAV1360	pKPC_UVA01	2	6454	1	6446	6446	6442	99.98
CAV1391	pKPC_UVA01	2	6454	1	6477	6477	6446	100
CAV1016	pKPC_UVA01	2	6454	1	6443	6443	6443	100
CAV1205	pKPC_UVA02	1	9038	1	9023	9023	9023	100
CAV1207	pKPC_UVA02	1	9038	1	9021	9021	9021	100
CAV1338	pKPC_UVA02	1	9038	1	9023	9023	9023	100
CAV1576	pKPC_UVA02	1	9038	1	9044	9044	9027	100
CAV1578	pKPC_UVA02	1	9038	1	9041	9041	9033	99.98
CAV1597	pKPC_UVA02	1	9038	1	9022	9022	9022	100
CAV1015	pKPC_UVA02	1	9038	1	9039	9039	9024	100
CAV1205	pKPC_UVA02	2	4041	1	4031	4031	4031	100
CAV1207	pKPC_UVA02	2	4041	1	4031	4031	4031	100
CAV1338	pKPC_UVA02	2	4041	1	4030	4030	4030	100
CAV1576	pKPC_UVA02	2	4041	2	4629	4030	4030	100
CAV1578	pKPC_UVA02	2	4041	2	4331	4030	4030	100
CAV1597	pKPC_UVA02	2	4041	2	4302	4029	4027	100
CAV1015	pKPC_UVA02	2	4041	1	4031	4031	4031	100

[a]This isolate does not contain pKPC_UVA01 according to the definition of presence presented in Figure 1 (≥90% of plasmid reference with read depth ≥10). However, part of the pKPC_UVA01 sequence is present (71%; see supplementary table 2), which is consistent with the PCR results.

Supplementary Table 4. Study and corresponding sequencing identifiers for data deposited in National Center for Biotechnology Information

Isolate name	Sequence id	Accession Number
***Escherichia coli* transformant**		
GH1016	C00016676	SRR1582854
GH1015	C00016712	SRR1582855
**Initial Clinical KPC- *Klebsiella pneumoniae* (KPC-*Kp*)**		
CAV1363	C00016732	SRR1582856
CAV1351	C00016760	SRR1582857
CAV1016	C00016749	SRR1582858
CAV1017	C00016765	SRR1582859
CAV1360	C00016721	SRR1582860
CAV1042	C00016755	SRR1582861
CAV1064	C00016723	SRR1582862
CAV1216	C00016726	SRR1582863
CAV1378	C00016752	SRR1582864
CAV1066	C00016769	SRR1582865
CAV1062	C00016773	SRR1582866
CAV1061	C00016775	SRR1582867
CAV1596	C00016777	SRR1582868
CAV1203	C00016747	SRR1582869
CAV1217	C00016738	SRR1582870
CAV1550	C00016767	SRR1582871
CAV1074	C00016724	SRR1582872
CAV1041	C00016725	SRR1582873

CAV1416	C00016735	SRR1582874
CAV1344	C00016722	SRR1582875
CAV1292	C00016727	SRR1582876
CAV1290	C00016729	SRR1582877
CAV1194	C00016734	SRR1582878
CAV1338	C00016754	SRR1582879
CAV1182	C00016771	SRR1582880
CAV1207	C00016748	SRR1582881
CAV1391	C00016753	SRR1582882
CAV1597	C00016758	SRR1582883
CAV1576	C00016764	SRR1582884
CAV1578	C00016770	SRR1582885
CAV1205	C00016776	SRR1582886
CAV1562	C00016778	SRR1582887
CAV1142	C00016731	SRR1582888
CAV1077	C00016761	SRR1582889
CAV1237	C00016762	SRR1582890
CAV1145	C00016733	SRR1582891
CAV1699	C00016774	SRR1582892
CAV1040	C00016751	SRR1582893
CAV1397	C00016763	SRR1582894
CAV1392	C00016766	SRR1582895
CAV1345	C00016736	SRR1582896
CAV1214	C00016757	SRR1582897
CAV1209	C00016759	SRR1582898
CAV1585	C00016768	SRR1582899
CAV1410	C00016737	SRR1582900

CAV1127	C00016742	SRR1582901
CAV1599	C00016746	SRR1582902

Supplementary table 5. Longitudinal Pairs of isolates from same patient of KPC-*Kp*

First Isolate	Second Isolate	Days Between Collection
CAV1041	CAV1040	1
CAV1216	CAV1397	275
CAV1351	CAV1392	32
CAV1338	CAV1345	9
CAV1182	CAV1214	95
CAV1205	CAV1209	18
CAV1576	CAV1585	8
CAV1391	CAV1410	114
CAV1062	CAV1127	266
CAV1596	CAV1599	3
